# Genetic dissection of seed-iron and zinc concentrations in chickpea

**DOI:** 10.1038/srep24050

**Published:** 2016-04-11

**Authors:** Hari D. Upadhyaya, Deepak Bajaj, Shouvik Das, Vinod Kumar, C. L. L. Gowda, Shivali Sharma, Akhilesh K. Tyagi, Swarup K. Parida

**Affiliations:** 1 International Crops Research Institute for the Semi-Arid Tropics (ICRISAT), Patancheru 502324, Telangana, India; 2 National Institute of Plant Genome Research (NIPGR), Aruna Asaf Ali Marg, New Delhi 110067, India; 3 National Research Centre on Plant Biotechnology (NRCPB), New Delhi 110012, India

## Abstract

The SNP-based high-resolution QTL mapping mapped eight major genomic regions harbouring robust QTLs governing seed-Fe and Zn concentrations (39.4% combined phenotypic variation explained/PVE) on six chromosomes of an intra-specific high-density genetic linkage map (1.56 cM map-density). 24620 SNPs discovered from genome-wide GBS (genotyping-by-sequencing) and 13 known cloned Fe and Zn contents-related chickpea gene-orthologs were genotyped in a structured population of 92 sequenced *desi* and *kabuli* accessions. The large-scale 16591 SNP genotyping- and phenotyping-based GWAS (genome-wide association study) identified 16 genomic loci/genes associated (29% combined PVE) with seed-Fe and Zn concentrations. Of these, 11 trait-associated SNPs in the genes linked tightly with eight QTLs were validated by QTL mapping. The seed-specific expression, including pronounced differential-regulation of 16 trait-associated genes particularly in accessions/mapping individuals with contrasting level of seed-Fe and Zn contents was apparent. Collectively, the aforementioned rapid integrated genomic strategy led to delineate novel functional non-synonymous and regulatory SNP allelic-variants from 16 known/candidate genes, including three strong trait-associated genes (encoding late embryogenesis abundant and yellow stripe-like 1 protein, and vacuolar protein sorting-associated protein) and eight major QTLs regulating seed-Fe and Zn concentrations in chickpea. These essential inputs thus have potential to be deployed in marker-assisted genetic enhancement for developing nutritionally-rich iron/zinc-biofortified chickpea cultivars.

The global population strength is fast rising in an alarming rate and anticipated to cross the mark of nine billion by 2050^1^,^2^. Ensuring food and nutritional security of this fast growing population will pose a huge challenge especially in the era of climatic variability and resource scarcity. Adequate intake of nutritious food enriched with essential micronutrients is prerequisite for humans to meet their metabolic need and maintain good health. However, over three billion people, including one third of the children in developing countries are suffering from micronutrient malnutrition or hidden hunger world-wide^3^,^4^,^5^. Henceforth, micronutrient malnutrition/hidden hunger is a major global health concern and require immediate attention^6^. Iron (Fe) and Zinc (Zn) are the major components of micronutrient and play a crucial role in growth and development of human by acting as co-factors for several proteins, including haemoglobin, cytochrome and transcription factors. The Fe and Zn deficiencies are reportedly the most widespread and commonly observed micronutrient deficiencies in human beings^7^,^8^,^9^. Fe and Zn deficiency leads to several physiological disorders, including anaemia, tissue hypoxia, impaired physical growth, hypogonadism, dwarfism, orificial and acral dermatitis. About half of the world population is at risk for inadequate intake of Fe and Zn in their diet^3^,^10^. Consequently, several strategies, including mineral supplementation, dietary diversification and food fortification have been implemented to mitigate the micronutrient deficiency/hidden hunger^4^,^11^. Nonetheless, these attempts achieved a limited success due to lack of proper cultural awareness and inappropriate socio-economic infrastructure^11^.

With the advancement of cutting edge next generation sequencing (NGS)- and array-based genotyping strategies, the genomics-assisted breeding, which primarily involves identification and introgression of useful genes/QTLs (quantitative trait loci) regulating Fe and Zn contents, can be deployed as a powerful intervention tool for biofortification of food crops^4^,^11^,^12^. The essential inputs obtained from the above-mentioned strategy will be useful to increase the bioavailable micronutrients and thus enhance the quality component traits in food crops for nutritional security. However, the accumulation of mineral nutrients, including Fe and Zn in the edible parts (mostly seeds/grains) of crops is tightly regulated through complex cellular and genetic mechanism. The proportionate quantity of Fe and Zn present in seeds is a complex quantitative trait and governed by many major genes/QTLs^13^,^14^. Therefore, it is highly imperative to dissect the genetic basis of variability governing Fe and Zn concentrations in seeds of food crops. Tremendous efforts have been made to delve deeper into the complex genetic architecture of this trait in several crop species, including cereals and legumes^2^,^5^,^6^,^7^,^8^,^9^,^10^,^11^,^12^,^13^,^14^,^15^,^16^,^17^,^18^,^19^. Among legumes with much emphasis on common bean, attempts have been made to decipher the underlying genetic factors regulating Fe and Zn concentrations in seeds of *Medicago*, lentil, field pea and chickpea^8^,^9^,^13^,^14^,^20^,^21^,^22^,^23^,^24^.

Chickpea is a self-pollinated, annual diploid and highly nutritious legume with a genome size of ~740 Mbp that is considered as a staple source of protein^25^,^26^. This legume crop ranks second in consumption and third in production among pulses grown world-wide^25^,^26^. The genomes of two chickpea cultivars (*desi* and *kabuli*) with contrasting agronomic traits representing diverse genepools, have been sequenced^27^,^28^,^29^. Chickpea seeds constitute several nutritional composition, including protein, carbohydrate and minerals like Fe and Zn^5^,^30^. Considering the importance of this crop as a dietary element across a major part of global population, especially in developing countries, it is desirable to understand the complex genetic inheritance pattern and gene regulatory function of seed-Fe and Zn concentrations in chickpea leading to their bio-fortification through marker-assisted genetic enhancement. To date, only one 1536 SNP markers-based trait association mapping study has been reported to identify the major genomic loci/genes regulating seed-Fe and Zn concentrations in chickpea^14^. Unfortunately, no informative markers tightly linked to the major QTLs/genes governing seed-Fe and Zn contents are available that could be deployed in marker-assisted genetic improvement of chickpea. An integrated genomic approach involving traditional QTL mapping, association analysis and differential gene expression profiling is currently the most efficient strategy for rapid dissection of complex yield and quality component quantitative traits in diverse crop plants, including chickpea^31^,^32^,^33^,^34^,^35^,^36^,^37^,^38^,^39^,^40^,^41^,^42^,^43^.

In this aspect, the current study has made an effort to integrate QTL mapping with genome-wide association study (GWAS), candidate gene-based association mapping and expression profiling using large-scale natural germplasm lines and mapping populations to delineate functionally relevant molecular tags (markers, genes, QTLs and alleles) regulating seed-Fe and Zn concentrations, with an ultimate objective of genetic enhancement in chickpea.

## Results and Discussion

### Genome-wide distribution of SNPs

**Figure 1:**
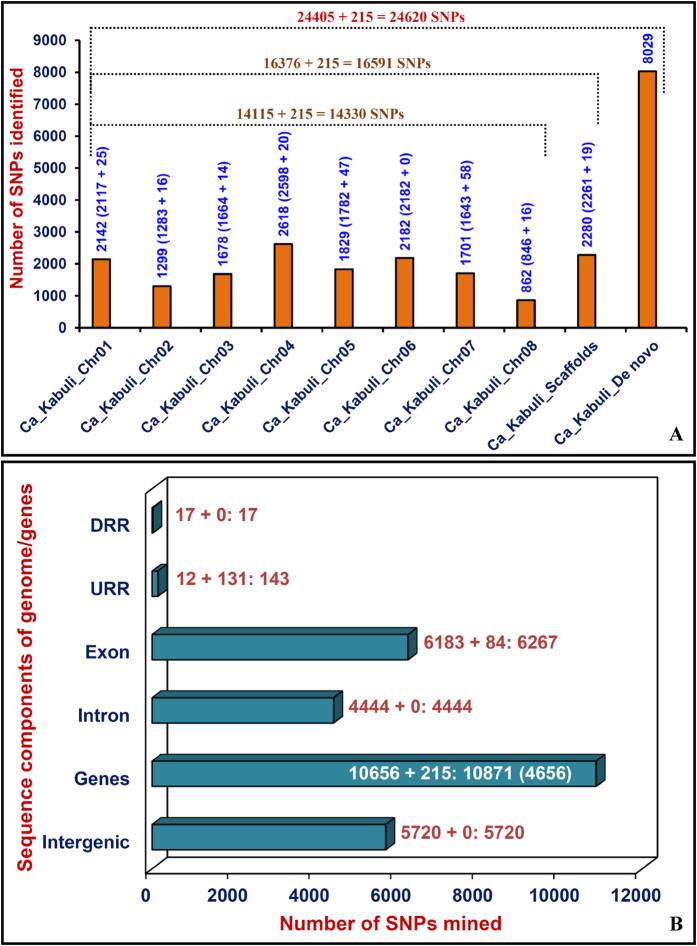
(**A**) Genomic distribution of 24620 SNPs discovered from the reference *kabuli* genome (eight chromosomes and scaffolds)- and *de novo*-based GBS (24405 SNPs) assays as well as by combining the Fe and Zn contents-related known cloned chickpea gene orthologs-led amplicon resequencing assay with a Illumina GoldenGate (215 SNPs) SNP genotyping assay. **(B)** Structural annotation of SNPs in the diverse non-coding (intron, URR and DRR) and coding (synonymous and non-synonymous) sequence components of genes and intergenic regions of *kabuli* genome. The CDS (coding sequences), URR (upstream regulatory region) and DRR (downstream regulatory region) of genes (mentioned in the Parentheses) were demarcated according to gene annotation information of reference *kabuli* genome (Varshney *et al*.^35^). The digits mentioned within/above the bars indicate the structural annotation of genome-wide GBS and candidate gene-based SNPs.

The *kabuli* reference genome (16376)- and *de novo* (8029)-based GBS (genotyping-by-sequencing) assays were employed to mine 24405 high-quality SNPs from 92 *desi* and *kabuli* chickpea accessions (Table S1)^44^. The GBS assay is advantageous in rapid large-scale mining and high-throughput genotyping of high-quality accurate genome-wide SNPs simultaneously for high-resolution genetic and association mapping^37^,^38^,^39^,^44^,^45^. Henceforth, this NGS-based strategy has potential to identify genes/QTLs regulating important agronomic traits in chickpea. A diverse array of known cloned genes involved in homeostasis, including uptake, translocation, regulation, signaling, storage and compartmentalization of Fe and Zn-contents have been isolated and characterized in multiple crop plants, including dicots^46^,^47^,^48^. Therefore, it would be interesting to assess the association potential of these known cloned genes for seed-Fe and Zn contents in chickpea. This can be simply achieved by large-scale genotyping of novel coding and regulatory SNP allelic variants mined from the seed-Fe and Zn contents-related known cloned genes in a diverse set of phenotypically well-characterized natural germplam lines. With a prime objective to discover gene-based SNPs, the high-quality cloned amplicon sequences of 13 known Fe and Zn content-regulating gene orthologs of chickpea (involved in transport and signalling pathways of Fe and Zn contents in crop plants) were compared initially among 12 representative *desi* and *kabuli* accessions. The subsequent high-throughput genotyping of these mined SNPs in all 92 *desi* and *kabuli* accessions by Illumina GoldenGate assay identified 215 coding and regulatory SNPs in 13 genes (46421 bp) with a mean density of one SNP per 215.9 bp (Table 1). Altogether, *kabuli* reference genome- and *de novo*-based GBS (24405) assay as well as known gene-derived (215) SNP genotyping assay discovered 24620 SNPs (Fig. 1A). This includes 16591 reference genome-based SNPs, of which 14330 and 2280 SNPs were physically mapped across eight chromosomes (mean map density: 24.2 kb) and unanchored scaffolds of *kabuli* genome, respectively.

The structural annotation of 16591 SNPs, including 16376 *kabuli* reference genome- and 215 gene-based SNPs was performed. This revealed presence of 5720 and 10871 SNPs in the intergenic regions and 4656 genes, respectively (Fig. 1B). The annotation of SNPs within the genes exhibited the occurrence of a highest and lowest number of 6267 (6183 genome-wide and 84 gene-based SNPs) and 17 SNPs in the exons/CDS (coding sequence) and DRRs (downstream regulatory regions), respectively. The coding SNPs comprised of 4259 synonymous and 2008 non-synonymous (missense and nonsense) SNPs. The regulatory SNPs (143, including 12 genome-wide and 131 gene-based SNPs) mostly derived from the URRs (upstream regulatory regions) of *kabuli* chickpea genes (Fig. 1B). The GBS-based genome-wide and known gene-derived novel SNP allelic variants discovered among a diverse set of *desi* and *kabuli* chickpea accessions can be deployed for various high-throughput genetic analysis particularly in rapid identification of genes (alleles)/QTLs governing complex Fe and Zn-content traits in chickpea.

### Construction of a high-density intra-specific chickpea genetic linkage map

**Figure 2:**
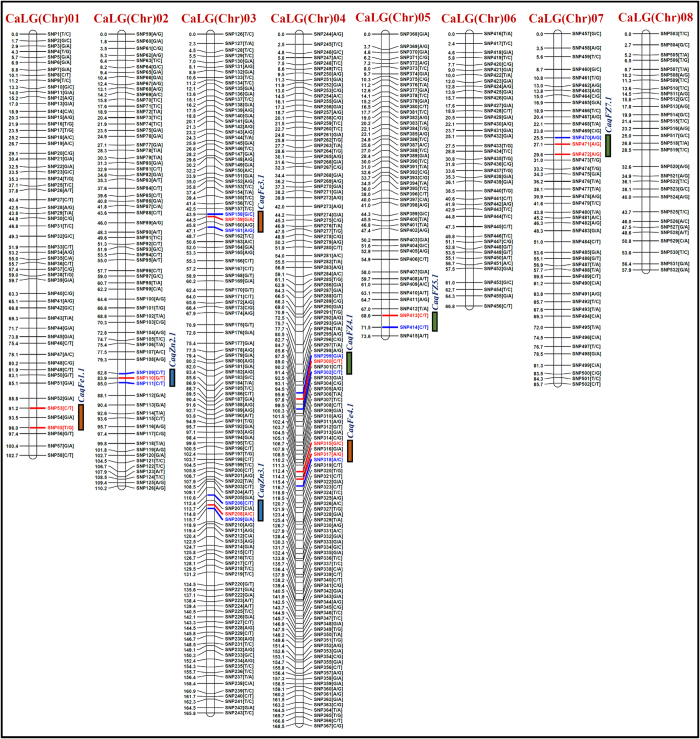
Eight major genomic regions harbouring eight robust QTLs (16.9–23.6% PVE) associated with seed-Fe and Zn concentrations were identified and mapped on six chromosomes (LOD > 6.0) using a 277 RIL mapping population (ICC 4958 × ICC 8261) of chickpea. The genetic distance (cM) and identity of the marker loci integrated on the chromosomes are indicated on the left and right side of the chromosomes, respectively. The markers flanking and tightly linked to the QTLs are indicated with blue and red coloured lines, respectively. Orange, blue and green boxes indicate the QTLs governing seed-Fe, seed-Zn and both seed-Fe and Zn concentrations, respectively mapped on the chromosomes of a high-density genetic map.

For generating an intra-specific high-resolution genetic linkage map, 536 SNPs (529 genome-wide GBS- and 7 known gene-based SNPs) exhibiting polymorphism between parental accessions (*desi* accession ICC 4958 and *kabuli* accession ICC 8261) were genotyped in 277 individuals of a RIL (recombinant inbred line) mapping population (ICC 4958 x ICC 8261). The use of these SNP genotyping data in linkage analysis mapped 533 SNPs onto eight LGs (linkage groups) of an intra-specific genetic map of chickpea (Table S2, Fig. 2). The genetic map consisting of eight LGs covered a total map length of 831.2 cM with a mean inter-marker distance of 1.56 cM. The LG4 had longest map length spanning 168.5 cM, whereas LG8 exhibited shortest map length of 57.9 cM (Table S2, Fig. 2). A highest number of SNPs were mapped on LG4 (124 markers), followed by LG3 (118) and lowest on LG8 (30). The most saturated genetic map was observed in LG4 (a mean inter-marker distance of 1.36 cM), while LG8 (1.93 cM) had the least saturated map (Table S2, Fig. 2). Overall, an intra-specific genetic linkage map constructed in our study had a comparable/higher mean map-density (an average inter-marker distance of 1.56 cM) than that recently documented in diverse microsatellite and SNP markers-anchored intra- and inter-specific genetic maps of chickpea^35^,^36^,^38^,^39^,^41^,^49^,^50^,^51^,^52^,^53^,^54^,^55^.

### Molecular mapping of QTLs associated with seed-Fe and Zn concentrations

**Figure 3:**
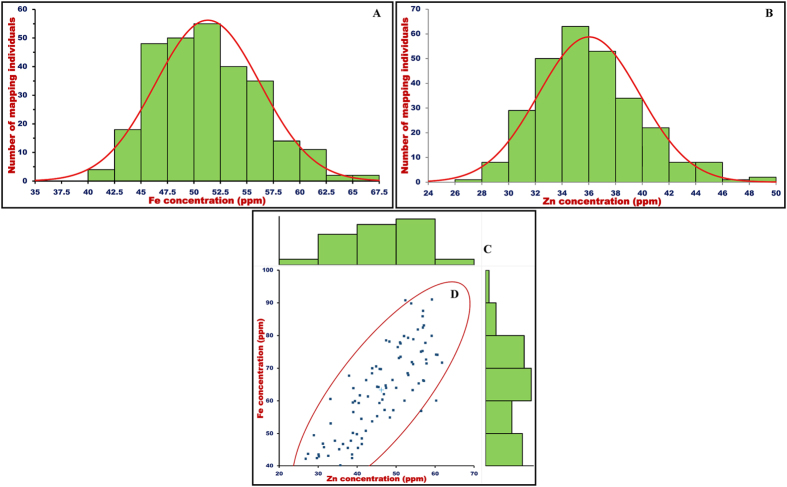
Frequency distribution of seed-Fe and Zn concentration traits variation estimated among 277 individuals and parental accessions of an intra-specific F_7_ RIL mapping population (ICC 4958 × ICC 8261) (**A** , **B**) as well as in a structured population of 92 *desi* and *kabuli* chickpea accessions **(C)** depicted a goodness of fit to the normal distribution. (**D**) Correlation based on Pearson’s coefficient estimated between seed-Fe and Zn concentrations among 92 chickpea accessions.

A highly significant difference (P < 0.001) of seed-Fe (51.3 ± 5.0, 40.3–67.0 ppm with 81% H^2^) and Zn (36.0 ± 3.8, 27.9–48.5 ppm with 83% H^2^) concentrations in two parental accessions and 277 individuals of a RIL mapping population [ICC 4958 (Fe: 43.0 ± 2.9 and Zn: 30.2 ± 2.5 ppm) × ICC 8261 (Fe: 64.8 ± 3.3 and Zn: 42.0 ± 2.7 ppm)] across two years based on ANOVA was observed (Table S3, Fig. 3A,B). Especially, ANOVA results inferred a highly significant difference (P < 0.001) among RIL individuals for both seed-Fe and Zn concentrations despite significant environmental (years and geographical locations) effects on these traits. A significant interaction between genotypes (G)/accessions and environment (E) for seed-Fe and Zn concentration traits was evident. However, G × E and E-variances (% of total mean squares) was found to be almost equal between seed-Fe and Zn concentrations at a significant level of P < 0.001. The normal frequency distribution, including bi-directional transgressive segregation of two target traits among mapping individuals and parental accessions was evident (Fig. 3A,B). The Pearson’s correlation coefficient estimation revealed a significant positive correlation (0.53–0.68 with mean r: 0.61, P < 0.0001) between seed-Fe and Zn contents among individuals of a mapping population.

The use of genotyping data of 533 SNPs mapped on an intra-specific genetic linkage map and phenotyping data of 277 RIL mapping individuals in QTL mapping identified eight major genomic regions underlying eight robust QTLs (6.0–8.8 LOD) significantly associated with seed-Fe and Zn concentrations in chickpea (Table 2, Fig. 2). These validated QTLs exhibiting consistent phenotypic expression (>10% phenotypic variation explained^49^) across environments (years and geographical locations) at a higher LOD (>6.0) and H^2^ (>90%) were considered as robust QTLs governing chickpea seed-Fe and Zn contents. Eight major genomic regions harbouring eight robust QTLs (*CaqFe1.1*, *CaqZn2*.1, *CaqFe3*.1 *CaqZn3*.1 *CaqFZ4*.1 *CaqFe4*.1 *CaqFZ5*.1 and *CaqFZ7*.1) were mapped on six chromosomes (except chromosomes 6 and 8) (Table 2, Fig. 2). These QTL intervals spanning in the range from 2.2 cM on chromosome 2 to 4.8 cM on chromosome 1 covered with a total of 27 mapped SNP loci. The proportion of phenotypic variation explained (PVE) by individual QTL ranged from 16.9 to 23.6%. The combined PVE measured for all eight robust QTLs was 39.4%.

For seed-Fe concentration, three major genomic regions harbouring three robust QTLs (21.1–23.4% PVE with 8.0–8.8 LOD) were mapped on three chromosomes (1, 3 and 4) (Table 2, Fig. 2). The combined PVE of all three robust QTLs was 34.6%. These QTL regions covered (3.2 cm on chromosome 3 to 4.8 cM on chromosome 1) with 11 SNPs, were genetically mapped on chromosomes. The identified major QTLs exhibited positive additive gene effect for increasing seed-Fe concentration with higher effective allelic contribution from ICC 8261. For seed-Zn concentration, two major genomic regions underlying two robust QTLs were identified and mapped on two chromosomes (2 and 3) with 18.7–21.8% PVE (7.0–8.3 LOD) (Table 2, Fig. 2). These QTL intervals covered (2.2 cM on chromosome 2 to 3.3 cM on chromosome 3) with seven SNPs, were genetically mapped on chromosomes. The combined PVE for all two robust QTLs was 33.9%. These QTLs revealed positive additive gene effect for increasing seed-Zn concentration with major allelic contributions from ICC 8261. For both seed-Fe and Zn concentrations, three major genomic regions harbouring three robust QTLs were identified and mapped on three chromosomes (4, 5 and 7) with 16.9–18.5% PVE (6.0–6.7 LOD) (Table 2, Fig. 2). The combined PVE of all three robust QTLs was 36.4%. These QTL regions covered (2.9 cm on chromosome 5 to 4.1 cM on chromosome 7) by nine genetically mapped SNP loci, were exhibited positive additive gene effect for increasing both seed-Fe and Zn concentrations with maximum effective allelic contribution from ICC 8261. All 11 informative SNP loci tightly linked to eight QTLs regulating seed-Fe and Zn concentrations are provided in the Table 2.

The 11 SNP loci linked to the robust eight major QTLs governing seed-Fe and Zn concentrations were compared/correlated with that of an only previous association mapping study on similar traits available till date in chickpea^14^. This revealed correspondence of two SNP loci (SNP53 and SNP317) in the genes (encoding elongation factor and I-Q domain like protein) linked tightly with two major QTLs (*CaqFe1.1* and *CaqFe4.1*) between previous and present study. Molecular mapping of QTLs associated with any of such traits pertaining to seed-Fe and Zn concentrations has not been reported so far in chickpea. In this context, the informative SNPs especially derived from the major known/candidate genes identified and mapped at robust seed-Fe and Zn concentrations-associated QTL regions in our study seem quite relevant. The gene-based SNPs tightly linked with major QTLs identified, can be utilized in marker-assisted genetic improvement for developing cultivars with high level of Fe and Zn contents in the seeds of chickpea.

### Association mapping of seed-Fe and Zn concentrations

The implication towards integrating GWAS with candidate gene-based association analysis for efficient quantitative dissection of diverse complex agronomic traits have been realized currently in chickpea^38^,^39^,^56^,^57^. Considering this, an integrated high-resolution association mapping strategy involving GWAS and candidate gene-based association analysis has been deployed in our study to narrow-down the potential genomic loci/genes governing quantitative traits of seed-Fe and Zn concentrations at a genome-wide scale in chickpea. For GWAS and candidate gene-based association mapping, the genotyping information of 16591 informative SNPs (differentiating the 92 *desi* and *kabuli* chickpea accessions) mined through genome-wide GBS (16376 SNPs)- and known/candidate gene (215 SNPs)-based SNP genotyping assays were utilized (Fig. 1A). The classification of 92 chickpea accessions into two distinct population groups (POP I and POP II) based on neighbour-joining phylogenetic tree, high-resolution population genetic structure and principal component analysis (PCA) using 16591 SNP genotyping data was apparent^37^. The determination of LD pattern using the genotyping data of 14330 genome-wide SNPs (physically mapped on eight *kabuli* chromosomes) in 92 *desi* and *kabuli* chickpea accessions exhibited 200–250 kb LD decay across chromosomes. The normal frequency distribution along with a wider phenotypic variation for seed-Fe (63.3 ± 13.3, 40.2–91.0 ppm with 80% H^2^) and Zn (46.2 ± 9.1, 26.8–61.8 ppm with 82% H^2^) concentrations among 92 *desi* and *kabuli* chickpea accessions was observed (Table S3, Fig. 3C). A higher significant positive Pearson correlation (0.71–0.86 with mean r: 0.80, P < 0.0001) between these two target traits in 92 chickpea accessions was apparent (Fig. 3D). By comparing the multi-location field phenotyping information of seed-Fe and Zn concentrations observed in our constituted natural and mapping populations, a broader target trait variation in 92 natural germplasm lines than that of 277 RIL individuals of an intra-specific mapping population (ICC 4958 × ICC 8261) was observed. This infers that the natural germplasm lines representing 16 diverse geographical regions of the world selected by us are rich in allelic diversity for seed-Fe and Zn content traits. Therefore, the screened germplasm lines can serve as a useful genetic resource for mining novel functional allelic variants to accelerate trait association mapping at a genome-wide and/or gene-level in chickpea. This will essentially expedite the identification of potential genes/alleles regulating seed-Fe and Zn concentrations in domesticated *desi* and *kabuli* chickpea.

**Figure 4:**
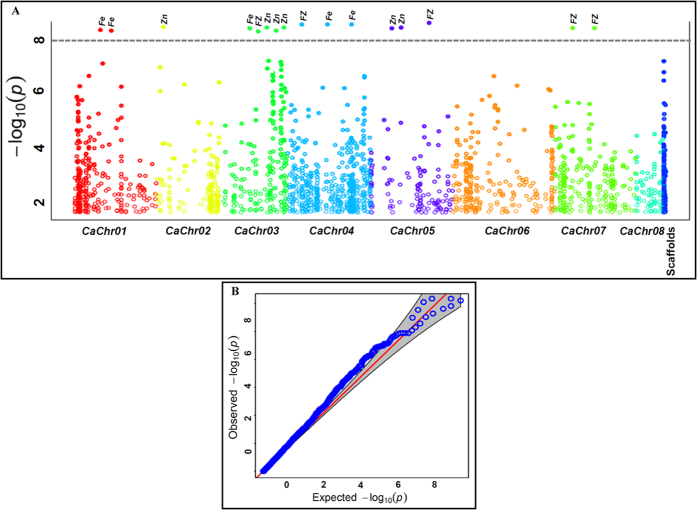
(**A**) GWAS-based Manhatton plot exhibiting significant P-values (measured by CMLM and P3D/EMMAX model) associated with seed-Fe and Zn concentrations using 16591 genome-wide GBS- and candidate gene-based SNPs in chickpea. The x-axis illustrates the relative density of reference genome-based SNPs physically mapped on eight chromosomes and scaffolds of *kabuli* genome. The y-axis displays the -log_10_ (P)-value for significant association of 16 SNP loci with seed-Fe and Zn concentrations. The SNPs revealing significant association with seed-Fe and Zn concentrations at cut-off P value ≤10^−8^ are marked with dotted lines. (**B**) Quantile-quantile plot illustrating the comparison between expected and observed -log_10_(P)-values with a FDR cut-off <0.05 to detect significant genomic loci (genes) associated with seed-Fe and Zn concentrations in chickpea.

The CMLM and P3D/EMMAX model-based association mapping approach (FDR cut-off ≤0.05) identified 16 genomic loci (gene-based SNPs) exhibiting significant association (P ≤ 10^−7^) with seed-Fe and Zn concentrations in chickpea (Table 3, Fig. 4). Twelve SNPs of these, were scanned from the genome-wide GBS information, whereas four SNPs derived from the known cloned *kabuli* chickpea genes regulating Fe and Zn contents. All 16 trait-associated SNPs were physically mapped on six chromosomes (except chromosomes 6 and 8) of *kabuli* genome (Table 3, Fig. 4). A highest number of five trait-associated genomic loci were mapped on chromosome 3. Nine and seven of 16 trait-associated genomic loci was represented from diverse coding (five synonymous and four non-synonymous SNPs) and non-coding (four intronic and three URRs) sequence components of 16 genes, respectively (Table 3, Fig. 4). The phenotypic variation for seed-Fe and Zn concentrations explained by 16 known/candidate gene-derived maximum effect genomic SNP loci varied from 6 to 14% (P: 1.2 × 10^−8^ to 1.0 × 10^−9^) among 92 chickpea accessions (Table 3, Fig. 4). The combined phenotypic variation for seed-Fe and Zn concentrations explained by all significant 16 genic SNP loci was 29% (Table 3, Fig. 4). Specifically, six gene-based SNP loci, including one of each non-synonymous and regulatory SNPs were significantly associated (6–12% R^2^ with P: 3.2 × 10^−8^ to 1.3 × 10^−9^) with seed-Fe concentration in chickpea (Table 3, Fig. 4). For seed-Zn concentration, six, including two non-synonymous genic SNPs revealing significant association (7–14% R^2^ with P: 1.0 × 10^−7^ to 1.0 × 10^−9^) with target trait were detected. In addition, four gene-derived SNPs (including one non-synonymous and two URR-SNPs) associated (6–12% R^2^ with P: 1.2 × 10^−7^ to 1.0 × 10^−9^) with both seed-Fe and Zn concentrations were identified (Table 3, Fig. 4). The non-synonymous coding and regulatory SNPs in the known/candidate genes associated with seed-Fe and Zn concentrations delineated by us combining both GWAS and candidate gene-based association mapping in our study have functional significance towards quantitative dissection of these complex seed quality component traits in chickpea. These inputs further can essentially be utilized for establishing the rapid marker-trait linkages and efficient identification of potential genes/QTLs governing seed-Fe and Zn concentrations in chickpea.

Interestingly, we scaled-down 11, including six, two and three genomic SNP loci in 11 known/candidate genes showing strong association with seed-Fe, Zn and both Fe-Zn concentrations, respectively based on association mapping. Interestingly, these gene-based SNP loci were also found to be tightly linked with the robust nine major QTLs governing respective seed-Fe and Zn content traits (detected by QTL mapping in the present study) in chickpea (Tables 2 and 3). Of these, strong association of SNPs derived from three known/candidate genes [encoding late embryogenesis abundant (LEA) protein (12% R^2^ with P: 2.3 × 10^−9^), vacuolar protein sorting-associated protein (14% R^2^ with P: 1.0 × 10^−9^) and yellow stripe-like 1 (*YSL1*) protein (12% R^2^ with P: 1.0 × 10^−9^)] localized at three major target robust QTLs (17.7–23.4% R^2^ at 6.5–8.8 LOD) governing seed-Fe, Zn and both Fe-Zn concentrations, respectively was evident (Tables 2 and 3). Henceforth, 11 known/candidate gene-derived SNPs validated by both association and QTL mapping, were further selected as potential candidates regulating seed-Fe and Zn concentrations in chickpea.

### Validation of trait-associated genes through expression profiling

**Figure 5:**
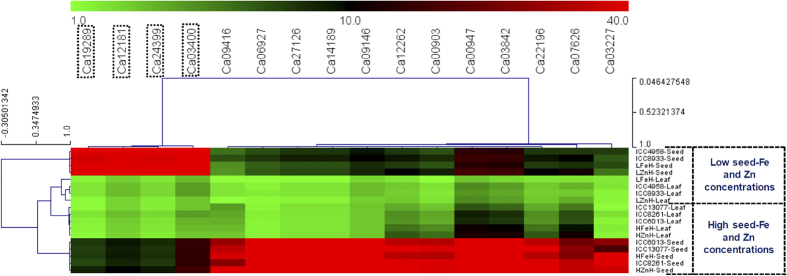
Hierarchical cluster display depicting the differential expression profiles of 16 seed-Fe and Zn concentrations-associated genes (including 11 validated by QTL mapping) with SNPs in the leaves and seed maturation developmental stages of five chickpea accessions/parents and four representative homozygous mapping individuals (ICC 4958 × ICC 8261) with contrasting level of low (ICC 4958 and ICC 8933) and high (ICC 8261, ICC 6013 and ICC 13077) seed-Fe and Zn concentrations. The average log signal expression value of genes in leaves and seed developmental stages are represented at the top with a colour scale; in which green, black and red color denote low, medium and high level of expression, respectively. The genes showing seed-specific expression, including pronounced differential down-regulation are highlighted with black boxes, respectively. The structural and functional annotation of 16 genes with SNPs are mentioned in the Table 3. The tissues/stages and genes utilized for expression profiling are provided on the right and upper side of expression map, respectively. Digits mentioned in the vertical and horizontal bars illustrate the range (minimum, optimum and maximum) of correlation coefficient varying among tissues/stages of diverse contrasting accessions/mapping individuals and across trait-associated genes, respectively. LFeH: low seed-Fe concentration homozygous mapping individual and LZnH: low seed-Zn concentration homozygous mapping individual. HFeH: high seed-Fe concentration homozygous mapping individual and HZnH: high seed-Zn concentration homozygous mapping individual.

A set of 16 trait-associated known/candidate genes with SNPs, including 11 validated by both QTL and association mapping were analysed to determine the differential expression profile and functional regulatory pattern of these genes governing seed-Fe and Zn concentrations in chickpea. The differential expression analysis (by quantitative RT-PCR assay) of these trait-associated genes in the leaves and seed maturation developmental stages of five chickpea accessions/parents and four homozygous mapping individuals with contrasting level of low (ICC 4958 and ICC 8933 with 42–43 ppm Fe and 27–30 ppm Zn) and high (ICC 8261, ICC 6013 and ICC 13077 with 65–90 ppm Fe and 42–62 ppm Zn) seed-Fe and Zn concentrations was performed. All 16 trait-associated known/candidate genes (11 genes validated by QTL mapping) exhibited seed-specific expression (>4-fold) as compared to leaf tissues of parental accessions and homozygous mapping individuals with low and high level of Fe and Zn contents (Fig. 5). Of these, twelve and four genes gave pronounced differential up (~7-fold, P ≤ 0.0001)- and down (~5-fold, P ≤ 0.0001)-regulation, respectively in the seeds of parental accessions/mapping individuals with high level of Fe and Zn concentrations as compared to that of parental accessions/individuals with low Fe and Zn contents (Fig. 5). Collectively, 16 differentially expressed potential genes with SNPs governing seed-Fe and Zn concentrations by integrating GWAS and candidate gene-based association analysis with expression profiling and 11 genes of these, by inclusion of QTL mapping with aforesaid strategy, were delineated in chickpea (Table 3).

The seed-specific expression, including a pronounced upregulation and enhanced transcript accumulation of three strong seed-Fe and Zn contents-associated known/candidate genes [encoding LEA, *YSL1* and vacuolar protein sorting-associated proteins] have ascertained significant involvement of these genes in storing high level of Fe and Zn contents in the maturing seeds of multiple crop (legumes) accessions^46^,^47^,^48^,^58^,^59^,^60^. These genes with useful properties of Fe/Zn-binding, are known to be involved in micronutrients trafficking, signalling and storage, especially overall homeostasis of Fe and Zn contents in seeds of crop plants. Recently, novel functional allelic variants scanned from the selected candidate genes that encode elongation factor, ubiquitin family [SWAP (suppressor-of-white-apricot)/Surp domain-containing protein] and IQ-domain like protein as well as unknown expressed protein, are found to be associated with seed-Fe and Zn concentrations based on 1536 SNP markers-led trait association mapping in chickpea^14^. A number of DUF 828 (domain of unknown function 828), WD40 [tryptophan-aspartic acid (W-D) dipeptide] repeat and RNA recognition motif domain-containing protein-coding genes belonging to Zn-finger family identified in our study are known to reflect molecular response towards Zn homeostasis in plant species^48^. The essential role of a diverse array of vacuolar protein sorting genes, including *HMA2*/*4* (heavy metal ATPases 2/4) (ATPase/P-type/H^+^ transporting proton pump) in regulation of seed Fe and Zn-contents suggests the critical involvement of these genes in translocation, remobilization and storage of Fe and Zn-contents in crop plants^61^. These vacuolar protein-encoding genes thus can be selected as potential candidates in deciphering the functional regulatory pathways underlying Fe and Zn content-accumulation in seeds of chickpea. Additionally, an *YSL* gene representing oligopeptide transporter OPT superfamily and ferritin (*FER*) gene regulating Fe translocation and storage, respectively have been isolated and characterized in higher plants^46^,^47^,^48^. A zinc/iron permease (*ZIP1*) gene encodes zinc/iron regulated transporter-related protein 1 belonging to a novel metal transporter ZIP gene family, is known to regulate transport of a wide-range of cations, including Fe and Zn for retaining their cellular homeostasis in plants^47^,^48^,^62^.

The aforementioned previous reports altogether suggests that the novel functional SNP allelic variants in the known/candidate genes and high-resolution major QTLs regulating seed-Fe and Zn concentrations delineated in our study, by deploying a combinatorial genomics-assisted breeding approach (QTL mapping, GWAS, candidate gene-based association analysis and differential expression profiling) seems much relevant towards rapid quantitative dissection of complex seed quality component target traits in chickpea. These informative molecular tags thus have potential to expedite marker-assisted genetic enhancement for developing iron/zinc-biofortified cultivars of chickpea.

## Methods

### Discovery and genotyping of SNPs

The structural/functional annotation, including genotyping information of *kabuli* reference genome- and *de novo*-based SNPs discovered from the sequenced 92 (39 *desi* and 53 *kabuli*) diverse chickpea accessions through a GBS-assay, were acquired^37^. For large-scale discovery and genotyping of gene-derived SNPs in chickpea, a selected set of 30 cloned genes known to be involved in regulation, transport and signalling pathways of Fe and Zn contents in crop plants, including dicots^46^,^47^,^48^ were obtained. To find out the best possible chickpea gene orthologs, the coding sequence of these known genes were BLAST homology searched (cut-off *E*-value ≤ 1e–41 and bit score ≥ 500) against the CDS of *kabuli* genes. The CDS and 2000-bp URRs of these identified chickpea gene orthologs were retrieved. These coding and regulatory sequences were used to design multiple overlapping forward and reverse primers with expected amplification product size of 400 to 500 bp per primer. The gene-based primers were amplified using the genomic DNA of 12 chickpea accessions (selected from aforesaid 92 accessions). The amplified PCR product was purified, cloned and sequenced twice in both forward and reverse directions by automated 96 capillary ABI 3730xl DNA Analyzer (Applied Biosystems, USA). The high-quality SNPs detected from the gene sequences were further genotyped in the genomic DNA of 92 chickpea accessions using Illumina GoldenGate assay. Subsequently, the genotyping information of SNPs generated among accessions was utilized for trait association mapping (following Bajaj *et al*.^41^).

### Phenotyping for seed-Fe and Zn contents

During normal crop season, 92 *desi* and *kabuli* chickpea accessions, and 277 individuals of an intra-specific F_7_ RIL mapping population (*desi* traditional cultivar/landrace ICC 4958 x *kabuli* landrace ICC 8261) were grown in the field (following randomised complete block design with at least two replications) for two consecutive years (2012 and 2013) at two diverse geographical locations (New Delhi: 28° 4′N/77° 2′E and Patancheru, Hyderabad: latitude 17° 3′N/longitude 77° 2′E) of India. The mature seeds of each accession and mapping individual (2–4 representative plants from each accession/individual) were collected separately during harvesting stage. The healthy mature seeds (20 g) of chickpea accessions were analyzed at Central Analytical Services Laboratory, ICRISAT, Patancheru to measure their Fe and Zn contents. Precautions were taken to avoid the contamination of seeds with dust and metal particles during harvest and while preparing them for analysis.

Further, the seed samples were washed with distilled water and oven-dried at 60 °C for 48 h before grinding. The dried-seed samples (20 g) from each accession were then powdered in a mill with Teflon chambers and ground samples kept overnight in an oven at 60 °C. The standards and samples were digested simultaneously with appropriate blanks in duplicate (two independent analyses). One g of ground sample was transferred to a digestion tube [75 ml capacity and 10 ml of tri-acid mixture consisting of nitric acid, sulfuric and perchloric acid in the ratio of 10:0.5:2 (v/v)] and the contents left overnight for cold digestion in a digestion chamber. The samples were digested initially at 120 °C for one h, followed by digestion at 230 °C for about 2 h to get clear and colourless digests. Following cooling of the digests, the contents were dissolved in distilled water and volume made up to 75 ml, and shaken well. Aliquots were taken from the digests and analyzed for Fe and Zn concentrations (mg kg^−1^) by atomic absorption spectrophotometry (Varian Spectra AA 20^63^). The concentrations of Fe and Zn were estimated and expressed as mg kg^−1^ (ppm) seed. The seed-Fe and Zn contents of accessions were measured on a plot basis and their mean values were used for statistical analysis.

The diverse statistical parameters, including mean, standard deviation, coefficient of variation (CV), analysis of variance (ANOVA), frequency distribution and Pearson’s correlation coefficient of seed-Fe and Zn concentrations among accessions and RIL mapping individuals were measured using SPSSv17.0 as per Bajaj *et al*.^41^. The inheritance pattern of seed-Fe and Zn concentration traits was determined by estimating the effect of genotypes (accessions) (G), environments (experimental years and/or geographical locations) (E) and G × E interactions based on two-way ANOVA. The broad-sense heritability [H^2^ = σ^2^g/(σ^2^g + σ^2^ge/n + σ^2^e/nr)] of these two target traits was measured individually using the optimum criteria such as σ^2^g (genetic), σ^2^ge (G × E) and σ^2^e (error) variance with n (number of experimental years/environments) =2 and r (number of replicates) =2.

### QTL mapping

The multi-location/years replicated field phenotyping data for seed-Fe and Zn contents (ppm) of parental accessions and 277 individuals derived from a RIL mapping population (ICC 4958 × ICC 8261) was obtained. A selected set of parental polymorphic GBS-based (obtained from Kujur *et al*.^37^) and known gene-derived SNPs physically mapped on eight chromosomes was genotyped in 277 RIL mapping individuals and parental accessions using an Illumina GoldenGate SNP genotyping assay (as per Bajaj *et al*.^41^). The SNP genotyping data of a RIL mapping population were utilized to construct an intra-specific high-density genetic linkage map. Subsequently, this was correlated with the aforementioned phenotyping data of RIL mapping individuals and parents for QTL mapping [composite interval mapping at significant LOD (logarithm of odds) threshold >4.0 at a p < 0.05] as per Bajaj *et al*.^41^ and Saxena *et al*.^42^. Accordingly, identification and molecular mapping of the major genomic regions underlying robust QTLs associated with seed-Fe and Zn concentrations were performed in chickpea.

### Association mapping

For GWAS, the information regarding the genetic diversity (phylogenetic tree, PCA and population structure) and genome-wide/population-specific LD patterns (LD estimates and LD decay) among 92 chickpea accessions were obtained from the previous study^37^. The population structure ancestry coefficient (Q), kinship matrix (K) and PCA (P) data as well as the genome-wide and candidate gene-based SNP genotyping and phenotyping information (seed-Fe and Zn concentrations) of 92 accessions were analysed through mixed model (P + K, K and Q + K)-based CMLM (compressed mixed linear model) and P3D (population parameters previously determined^64^)/EMMAX (efficient mixed model association expedited^65^) approaches of GAPIT (following^38^,^66^). To ascertain the accuracy and robustness of SNP marker-trait association, the comparison of observed and expected -log_10_(P)-value relative distribution for each trait-associated genomic loci based on quantile-quantile plot and correction of their adjusted P-value threshold of significance for multiple comparison by false discovery rate (FDR cut-off ≤0.05, Benjamini and Hochberg 1995) were performed following Kujur *et al*.^38^ and Kumar *et al*.^66^. The degree of association of SNP loci with seed-Fe and Zn concentration traits was estimated by the R^2^ using a model with the SNP and adjusted P-value following a FDR-controlling method. The SNP loci in the genomic (gene) regions revealing significant association with seed-Fe and Zn concentrations at the lowest FDR adjusted P-values (threshold P < 10^−7^) and highest R^2^ (degree of SNP marker-trait association) were identified in chickpea.

### Expression profiling

To determine the regulatory pattern of candidate genes associated (validated by QTL mapping and association analysis) with seed-Fe and Zn concentrations, the differential expression profiling of these genes was performed using the quantitative RT-PCR assay. The leaves and late seed maturation developmental stages [21–30 days after podding (DAP)^67^] of five chickpea accessions as well as parents and four homozygous individuals of a RIL mapping population (ICC 4958 × ICC 8261) with contrasting level of low and high seed-Fe and Zn contents (ppm), were used for RNA isolation. In the RT-PCR assay, RNA isolated from three independent biological replicates of each sample and two technical replicates of each biological replicate with no template and primer as control were utilized. In addition, an internal control gene elongation factor 1-alpha (*EF1α*) for normalization of expression value and gene-specific primers for determining the differential gene expression profile across tissues of accessions and mapping parents/individuals were used (as per Bajaj *et al*.^67^). The significant difference of gene expression among accessions and mapping parents/individuals was determined by constructing a heat map through TIGR MultiExperiment Viewer (MeV, http://www.tm4.org/mev).

## Additional Information

**How to cite this article**: Upadhyaya, H. D. *et al*. Genetic dissection of seed-iron and zinc concentrations in chickpea. *Sci. Rep*. **6**, 24050; doi: 10.1038/srep24050 (2016).

## Supplementary Material

Supplementary Information

## Figures and Tables

**Table 1:** Thirteen chickpea gene orthologs known to regulate signalling and transport of Fe and Zn contents in crop plants selected for trait association mapping.

Characterized known cloned genes				*Kabuli* chickpea gene homologs			
Cropplants	Gene identities with functions		Geneaccession IDs	Geneaccession IDs	Chromosomes	Sequenced geneamplicon size (bp)	Coding and upstreamregulatory SNPs mined
*Arabidopsis*	*AtNRAMP1*	Natural resistance-associated macrophage protein 1	*At1g80830*	Ca02923	*Ca_Kabuli_Chr01*	3485	13
*Arabidopsis*	*AtZIP2*	Zinc/iron permease Zinc/iron regulated transporter-related protein 2	*At5g59520*	Ca02630	*Ca_Kabuli_Chr01*	3005	12
*Arabidopsis*	*AtFER3*	Ferritin 3	*At3g56090*	Ca12453	*Ca_Kabuli_Chr02*	2765	16
*Arabidopsis*	*AtFER1*	Ferritin 1	*At5g01600*	Ca09416	*Ca_Kabuli_Chr03*	2777	14
*Arabidopsis*	*AtYSL1*	Yellow stripe-like 1 Oligopeptide transporter OPT superfamily	*At4g24120*	Ca03842	*Ca_Kabuli_Chr04*	3923	20
*Arabidopsis*	*AtIRT3*	Iron-regulated transporter 3	*At1g60960*	Ca01633	*Ca_Kabuli_Chr05*	3224	15
*Arabidopsis*	*AtZIP1*	Zinc/iron permease Zinc/iron regulated transporter-related protein 1	*At3g12750*	Ca07626	*Ca_Kabuli_Chr05*	3044	14
*Arabidopsis*	*AtYSL2/3*	Yellow stripe-like 2/3 Oligopeptide transporter OPT superfamily	*At5g24380*	Ca04020	*Ca_Kabuli_Chr05*	3863	18
*Arabidopsis*	*AtHMA2/4*	Heavy metal ATPases 2/4 ATPase, P-type, H^+^ transporting proton pump	*At4g30110 At2g19110*	Ca03227	*Ca_Kabuli_Chr07*	5099	25
*Arabidopsis*	*AtNRAMP3*	Natural resistance-associated macrophage protein 3	*At2g23150*	Ca09322	*Ca_Kabuli_Chr07*	3533	12
*Arabidopsis*	*AtFRO2*	Ferric chelate reductase 2 FAD-binding 8	*At1g01580*	Ca13723	*Ca_Kabuli_Chr07*	4151	21
*Arabidopsis*	*AtNRAMP4*	Natural resistance-associated macrophage protein 4	*At5g67330*	Ca02191	*Ca_Kabuli_Chr08*	3521	16
*Arabidopsis*	*AtYSL4/6*	Yellow stripe-like 4/6 Oligopeptide transporter OPT superfamily	*At5g41000 At3g27020*	Ca27338	*Ca_Kabuli_Scaffold637*	4031	19

**Table 2:** Significant QTLs associated with seed-iron and zinc concentrations identified and mapped on the chromosomes of a high-density intra-specific chickpea genetic linkage map (ICC 4958 × ICC 8261).

QTLs	LGs/chromosomes	SNP marker intervals with geneticpositions (cM)	Traits associated	SNP markers tightlylinked with QTLs	2012			2013		
					LOD	PVE(%)	A	LOD	PVE(%)	A
*CaqFe1.1*	*Ca_Kabuli_Chr01*	SNP53(91.2)-SNP55(96.0)	Seed-Fe concentration	SNP53 and SNP55	8.6	23.6	6.5	8.5	22.6	6.8
*CaqZn2.1*	*Ca_Kabuli_Chr02*	SNP109(82.8)-SNP111(85.0)	Seed-Zn concentration	SNP110	8.3	21.8	5.4	8.2	20.5	5.5
*CaqFe3.1*	*Ca_Kabuli_Chr03*	SNP158(43.9)-SNP161(47.1)	Seed-Fe concentration	SNP159	8.5	20.8	4.8	8.0	21.1	4.3
*CaqZn3.1*	*Ca_Kabuli_Chr03*	SNP206(112.4)-SNP209(115.7)	Seed-Zn concentration	SNP208	7.3	19.6	5.1	7.0	18.7	5.5
*CaqFZ4.1*	*Ca_Kabuli_Chr04*	SNP299(87.5)-SNP302(91.4)	Seed-Fe and Zn concentration	SNP300	6.5	17.7	4.3	6.7	18.5	4.7
					7.1	18.6	4.1	7.4	17.8	4.0
*CaqFe4.1*	*Ca_Kabuli_Chr04*	SNP315(106.7)-SNP318(110.2)	Seed-Fe concentration	SNP315 and SNP317	8.8	22.6	6.7	8.6	23.4	6.5
*CaqFZ5.1*	*Ca_Kabuli_Chr05*	SNP413(68.6)-SNP414(71.5)	Seed-Fe and Zn concentration	SNP413	7.1	19.5	7.4	7.3	18.6	7.0
					6.8	23.7	8.3	7.6	20.5	8.0
*CaqFZ7.1*	*Ca_Kabuli_Chr07*	SNP470(25.5)-SNP472(29.6)	Seed-Fe and Zn concentration	SNP471 and SNP472	6.3	16.9	8.5	6.0	17.8	8.7
					6.5	19.5	8.3	6.3	18.9	8.4

^*^CaqFe1.1 (*Cicer arietinum* QTL for seed-iron concentration on chromosome 1 number 1), *CaqZn2.1* (*Cicer arietinum* QTL for seed-zinc concentration on chromosome 2 number 1) and *CaqFZ4.1* (*Cicer arietinum* QTL for seed-iron and zinc concentrations on chromosome 4 number 1). PVE: Phenotypic variation explained. A: Additive effect of alleles from ICC 8261 with high seed-iron and zinc concentrations. Details regarding SNPs are provided in the Table S1.

**Table 3:** Sixteen genes with SNPs regulating seed-Fe and Zn concentrations delineated by integrating GWAS and candidate gene-based association analysis with QTL mapping and expression profiling.

SNP IDs	*Kabuli*chromosomes	SNP physical positions (bp)	SNPs	Gene accession IDs	Structural annotation	Known/putative functions	Association analysis		
							P	R^2^ (%)	Traits associated
*^,^#CakSNP1426/SNP53	*Ca_Kabuli_Chr01*	16379258	[C/T]	Ca06927	Intron	Ubiquitin-associated/translation elongation factor EF1B	1.5 × 10^−8^	10	Seed Fe concentration
*^,^#CakSNP1628/SNP55	*Ca_Kabuli_Chr01*	24071014	[T/G]	Ca19289	Intron	AUX/IAA (auxin/indole-3-acetic acid) protein	1.9 × 10^−8^	10	Seed Fe concentration
*^,^#CakSNP14941/SNP110	*Ca_Kabuli_Chr02*	96532	[G/T]	Ca27126	Intron	Vacuolar protein sorting-associated protein	1.0 × 10^−9^	14	Seed Zn concentration
*^,^#SNP159	*Ca_Kabuli_Chr03*	20101590	[G/A]	Ca09416	URR	Ferritin 1 (*FER1*)	2.0 × 10^−8^	10	Seed Fe concentration
#CakSNP4409/ SNP186	*Ca_Kabuli_Chr03*	30837284	[C/T]	Ca12181	CDS(Nsyn)	Domain of unknown function 828 (*DUF828*)	1.2 × 10^−7^	6	Seed Fe and Zn concentrations
*^,^#CakSNP4476/SNP208	*Ca_Kabuli_Chr03*	31631451	[A/C]	Ca12262	CDS(Nsyn)	RNA recognition motif domain	2.0 × 10^−8^	12	Seed Zn concentration
#CakSNP4678/SNP216	*Ca_Kabuli_Chr03*	36050319	[T/C]	Ca00903	CDS(Syn)	SWAP (suppressor-of-white-apricot)/Surp domain-containing protein	1.2 × 10^−7^	9	Seed Zn concentration
#CakSNP4740/SNP229	*Ca_Kabuli_Chr03*	36387718	[C/T]	Ca00947	CDS(Syn)	WD40 [tryptophan-aspartic acid (W-D) dipeptide] repeat-containing protein	1.0 × 10^−7^	8	Seed Zn concentration
*^,^#SNP300	*Ca_Kabuli_Chr04*	4138693	[C/T]	Ca03842	URR	Yellow stripe-like 1 (*YSL1*)	1.0 × 10^−9^	12	Seed Fe and Zn concentrations
*^,^#CakSNP6693/SNP315	*Ca_Kabuli_Chr04*	30258596	[G/C]	Ca14189	CDS(Syn)	Late embryogenesis abundant (LEA) protein	1.3 × 10^−9^	12	Seed Fe concentration
*^,^#CakSNP7405/SNP317	*Ca_Kabuli_Chr04*	44814982	[A/G]	Ca09146	Intron	IQ-domain like protein	2.5 × 10^−9^	11	Seed Fe concentration
#CakSNP15585/SNP380	*Ca_Kabuli_Chr05*	43372	[C/T]	Ca22196	CDS(Syn)	Ubiquitin	1.1 × 10^−7^	8	Seed Zn concentration
#CakSNP15145/SNP396	*Ca_Kabuli_Chr05*	82342	[C/T]	Ca24399	CDS(Nsyn)	Unknown expressed gene	1.3 × 10^−7^	7	Seed Zn concentration
*^,^#CakSNP9048/SNP413	*Ca_Kabuli_Chr05*	41080035	[C/T]	Ca07626	CDS(Syn)	Zinc/iron permease (*AtZIP1*)	1.0 × 10^−8^	11	Seed Fe and Zn concentrations
*^,^#CakSNP11677/SNP471	*Ca_Kabuli_Chr07*	781409	[A/G]	Ca03400	CDS(Nsyn)	Octicosapeptide/Phox/Bem1p (PB1) domain-containing protein	3.2 × 10^−7^	6	Seed Fe concentration
*^,^#SNP472	*Ca_Kabuli_Chr07*	2515209	[A/G]	Ca03227	URR	Heavy metal ATPases 2/4 (*HMA2*/4)	3.5 × 10^−7^	10	Seed Fe and Zn concentrations

^*^Validated by QTL mapping and

^#^differential expression profiling.
